# On the Molecular Selection of Exopolysaccharide-Producing Lactic Acid Bacteria from Indigenous Fermented Plant-Based Foods and Further Fine Chemical Characterization

**DOI:** 10.3390/foods12183346

**Published:** 2023-09-06

**Authors:** Angel Angelov, Aneliya Georgieva, Mariana Petkova, Elena Bartkiene, João Miguel Rocha, Manol Ognyanov, Velitchka Gotcheva

**Affiliations:** 1 Department of Biotechnology, University of Food Technologies, 26 Maritza Blvd., 4000 Plovdiv, Bulgaria; gotcheva_v@uft-bio.com; 2 Institute of Food Preservation and Quality, 154 Vasil Aprilov Blvd., 4000 Plovdiv, Bulgaria; a.georgieva@uft-bio.com; 3 Department of Microbiology and Ecological Biotechnologies, Agricultural University, 12 Mendeleev Blvd., 4000 Plovdiv, Bulgaria; mpetkova@au-plovdiv.bg; 4 Department of Food Safety and Quality, Faculty of Veterinary, Lithuanian University of Health Sciences, Tilzes Str. 18, LT-47181 Kaunas, Lithuania; elena.bartkiene@lsmu.lt; 5 Faculty of Animal Sciences, Institute of Animal Rearing Technologies, Lithuanian University of Health Sciences, Tilzes Str. 18, LT-47181 Kaunas, Lithuania; 6 Universidade Católica Portuguesa, CBQF—Centro de Biotecnologia e Química Fina—Laboratório Associado, Escola Superior de Biotecnologia, Rua Diogo Botelho 1327, 4169-005 Porto, Portugal; jmfrocha@fc.up.pt; 7 LEPABE—Laboratory for Process Engineering, Environment, Biotechnology and Energy, Faculty of Engineering, University of Porto, Rua Dr. Roberto Frias, s/n, 4200-465 Porto, Portugal; 8 ALiCE—Associate Laboratory in Chemical Engineering, Faculty of Engineering, University of Porto, Rua Dr. Roberto Frias, s/n, 4200-465 Porto, Portugal; 9 Laboratory of Biologically Active Substances, Institute of Organic Chemistry with Centre of Phytochemistry, Bulgarian Academy of Sciences, 139 Ruski Blvd., 4000 Plovdiv, Bulgaria; manol.ognyanov@orgchm.bas.bg

**Keywords:** exopolysaccharides, lactic acid bacteria, *Lactiplantibacillus plantarum*, fermentation, carbohydrates, plant-based products

## Abstract

Exopolysaccharides (EPSs) produced by lactic acid bacteria present a particular interest for the food industry since they can be incorporated in foods via in situ production by selected starter cultures or applied as natural additives to improve the quality of various food products. In the present study, 43 strains were isolated from different plant-based fermented foods and identified by molecular methods. The species found were distinctively specific according to the food source. Only six *Lactiplantibacillus plantarum* strains, all isolated from sauerkraut, showed the ability to produce exopolysaccharide (EPS). The utilization of glucose, fructose and sucrose was explored with regard to EPS and biomass accumulation by the tested strains. Sucrose was clearly the best carbon source for EPS production by most of the strains, yielding up to 211.53 mg/L by strain *Lactiplantibacillus plantarum* ZE2, while biomass accumulation reached the highest levels in the glucose-based culture medium. Most strains produced similar levels of EPS with glucose and fructose, while fructose was utilized more poorly for biomass production, yielding about 50% of biomass compared to glucose for most strains. Composition analysis of the EPSs produced by strain *Lactiplantibacillus plantarum* ZE2 from glucose (EPS-1) and fructose (EPS-2) revealed that glucose (80–83 mol%) and protein (41% *w*/*w*) predominated in both analyzed EPSs. However, the yield of EPS-1 was twice higher than that of EPS-2, and differences in the levels of all detected sugars were found, which shows that even for the same strain, EPS yield and composition vary depending on the carbon source. These results may be the basis for the development of tailored EPS-producing starter cultures for food fermentations, as well as technologies for the production of EPS for various applications.

## 1. Introduction

The ability of lactic acid bacteria (LAB) to ferment the rich variety of carbohydrates in food raw-materials determines their key role in the food industry for the production of a variety of fermented products of plant and animal origin [1,2,3,4,5,6,7]. Fermented foods make up about a third of the human diet [8,9], and lactic acid fermentation, in particular, is one of the oldest approaches to improve the nutritional value and organoleptic characteristics of raw-materials, to extend their shelf-life and save energy in the preparation of plant-based foods, including bread and other baking goods [10,11,12,13].

Due to the biological activity of LAB and their enzymatic systems, a number of biochemical processes occur during fermentation, modifying the raw materials and leading to the production of foods with pleasant tastes, specific aromas and textures [14,15,16,17,18,19]. The use of lactic acid starter cultures enables the production of a wide range of products of animal and plant origin. In the past 15 years, the approach to replace some raw materials of animal origin with plant-based ones has been used to overcome the health limitations that exist for people with specific conditions, such as lactose deficiency or other specific dietary requirements [20].

LAB have been identified by the North American Food and Drug Administration (FDA) as generally regarded as safe (GRAS) organisms, and in the European Union (EU), the European Food Safety Authority (EFSA) has granted them a Qualified Presumption of Safety (QPS) status due to their proven long history of safe use. This makes them the preferred microbial agents in an extensive variety of food applications [21,22,23]. As an example, certain lactic acid bacteria are used for the preparation of sourdough-based products. They exert a positive effect on dough rising, which is comparable to that of the baker’s yeast, and the metabolites produced during the fermentation have an important role in the formation of a specific taste and aroma of the bakery products [24,25,26,27,28]. Homofermentative LAB produce lactic acid as a major final metabolite, whereas heterofermentative LAB release lactic acid, acetic acid or ethanol, and carbon dioxide during fermentation, which increases the loaf volume of the bread. They also increase the dough’s acidity and give the bakery products a specific flavor [29].

Homo- and heterofermentative lactobacilli attract a specific interest for the development of sourdough starter cultures due to their intrinsic resistance to high acidity, at which most of the other microorganisms in foodstuffs do not survive [30,31]. In this way, LAB promote a natural selection of desirable microbiota, in food matrixes, and the vanishing of spoilage microorganisms, thus contributing to the food safety. Strains of the *Lactobacillus sanfranciscensis* species are especially promising for such applications [32].

Under appropriate conditions, fruits and vegetables are subject to spontaneous lactic acid fermentation by LAB, which are naturally present in the raw materials. The processing conditions, the production environment and the other ingredients used also influence the composition of the fermenting microbiota. Homofermentative LAB, which ferment free sugars to lactic acid, take precedence in the production of fermented pickles, which are characterized by a pleasant lactic acid taste and aroma and a long shelf-life. LAB are also known for their ability to produce antimicrobial compounds, such as bacteriocins or bacteriocin-like compounds, organic acids and hydroperoxide, which leads to a biopreservation effect [33,34,35,36,37,38,39]. A nisin-type bacteriocin produced by *Lactococcus lactis* subsp. *lactis* has been used for decades as a biopreservative and as a biological control agent for some pathogens from the genera *Aeromonas*, *Bacillus*, *Clostridium*, *Enterococcus*, *Listeria*, *Micrococcus* and *Staphylococcus* [40,41,42]. Specifically selected LAB strains are also used as probiotics or in the composition of various probiotic products together with other microorganisms [43,44]. So far, the main genera from which strains with probiotic properties have been reported are *Lactobacillus*, *Lactococcus*, *Streptococcus*, *Enterococcus* and *Bifidobacterium*. When ingested in sufficient numbers, the probiotic strains beneficially affect the human resident microbiota and exert various positive health effects [45,46].

Most LAB, especially *Lacticaseibacillus*, *Lactiplantibacillus*, *Lactobacillus*, *Lactococcus*, *Lactilactobacillus*, *Lentilactobacillus*, *Leuconostoc*, *Limosilactobacillus*, *Pediococcus*, *Streptococcus* and *Weissella* species, are capable of synthesizing a variety of exopolysaccharides [47,48,49]. Their production is associated with the bacterial response to various stress biotic and abiotic stress factors (competition, pH, temperature, etc.) [50]. Exopolysaccharides are long-chain extracellular polysaccharides that are excreted into the medium space or remain bound to the bacterial cell surface. They have a positive effect on the textural properties of foods and other commercial products, such as pharmaceuticals. In the food industry, they are used as thickeners, stabilizers, emulsifiers, sweeteners and gelling agents [51,52]. Compared to other food texturizing additives, a key advantage of EPS produced by lactic acid bacteria is that their synthesis by selected starter cultures may occur in situ in the foods during the fermentation process, which lead to better rheological effects compared to EPS addition as an ingredient [50]. EPS from lactic acid bacteria are also known to exert various documented pro-health properties, including anti-cancer, antioxidant, prebiotic, immune-modulating, cholesterol-lowering, anti-inflammatory and other effects [53,54]. Four hundred different EPS variants with various chemical structures have been published, of which some can be linked to specific strains [55]. It is known that the main determinants of EPS synthesis are the culture conditions: growth medium composition, temperature, pH, duration, etc. [56,57]. However, the first and foremost factor is the growth medium composition as it directly affects EPS yield and chemical composition. LAB can produce EPS with broad-varying structures, depending on the strain and the carbon source in the medium [58]. On the other hand, the optimal composition of the growth medium and the choice of a suitable carbon source have an economic aspect.

In view of the numerous application possibilities related to EPS-producing lactic acid bacteria and the continuous search of commercial strains with such ability, the aim of the present study was to select exopolysaccharide-producing lactic acid bacteria isolated from different plant-based foods produced by spontaneous fermentation and characterize the EPS produced in culture media with different carbon sources.

## 2. Materials and Methods

### 2.1. Fermented Foods and Drinks

In the present study, 6 fermented food products of plant origin were sampled for the isolation of lactic acid bacteria: 4 traditional Bulgarian fermented products “Boza” (a rye boza, a rye-wheat boza and 2 products of wheat boza) purchased from the market, one home-made sauerkraut and a traditional fermented product from Benin—Dèguè, prepared from white sorghum (supplied by Prof. Innocent Bokossa-Yaou, Laboratoire de Microbiologie et des Technologies Alimentaires, Université d’Abomey-Calavi, Benin).

The sauerkraut used in our study was prepared in the lab according to a home recipe. White cabbage in a mature state was cleaned of the core and outer leaves. The cabbage was then placed in a 60 L container in brine prepared using water and NaCl (30 g/L). The closed container was incubated at 15–20 °C for 40 days.

All sampled foods were produced through natural fermentation and no starter cultures were applied in any of them.

### 2.2. Isolation and Characterization of Lactic Acid Bacteria from Plant-Based Fermented Foods

LAB isolates were obtained by using the reference ISO 15214:1998 method [59]. From each food product, a 10 g sample was homogenized with 90 mL of sterile peptone water, serially diluted and plated on the surface of de Man, Rogosa and Sharpe (MRS) agar supplemented with cycloheximide (Merck KGaA, Darmstadt, Germany). The plates were incubated under anaerobic conditions (AnaeroGen, Oxoid Ltd., Hampshire, UK) at 37 °C for 48 h, and enumeration of colony forming units (CFU) was performed. A number of colonies equal to the square root of the total number recorded in Petri dishes with 15 to 300 CFU were randomly selected for isolation. Single bacterial colonies with different morphology were individually selected for purification on MRS plates. Stock cultures were stored in Microbank™ vials (Pro-Lab Diagnostics Inc., Richmond Hill, ON, Canada) at −70 °C in 5 % (*v*/*v*) aqueous glycerol.

The initial characterization of the isolates was based on culture observations, cell morphology and biochemical tests. Cell morphology was examined using microscope (Laboval 4, Carlzeiss, Jena, Germany). Each bacterial isolate was Gram-stained, and only the Gram-positive ones were further tested for catalase production by placing a drop of 3% (*v*/*v*) hydrogen peroxide on the bacterial biomass. Based on the results, only Gram-positive, catalase-negative, nonmotile rods and cocci (LAB isolates) were further subjected to molecular identification.

### 2.3. DNA Extraction

The total genomic DNA was extracted from overnight cultures of the LAB isolates grown in MRS broth (Oxoid Ltd., Hampshire, UK). Biomass was washed twice and resuspended in 100 μL of DNase-free deionized water. DNA extraction was conducted using HigherPurity™ Bacterial Genomic DNA Isolation Kit (Canvax Biotech, S.L., Cordoba, Spain) according to the manufacturer’s instructions. Purification was performed by adding 20 µL of egg white lysozyme (Sigma Aldrich, St. Louis, MO, USA), 10 µL of proteinase K and 5 µL of ribonuclease A 10 mg/mL (Thermo Fisher Scientific Inc., Waltham, MA, USA) to the bacterial suspensions. The quality and concentration of the obtained DNA extracts were assessed through determination of absorbance at the wavelengths of 260 and 280 nm (Shimadzu UV-VIS, Shimadzu Corporation, Kyoto, Japan). The concentration of the obtained DNA extracts was calculated at 30–70 ng/mL. For the quality assessment, genomic DNA from the isolated LAB strains was mixed with a DNA loading buffer and the samples were placed in the wells of the 0.8% agarose gel. The gel electrophoresis system (VWR, Darmstadt, Germany) was powered with a DC voltage of 100 V and the separation process lasted for 40 min.

### 2.4. Molecular Identification of Bacteria by Sequencing of 16S rRNA

LAB identification was performed through polymerase chain reaction (PCR) amplification of the 16S rRNA gene with conventional PCR (2720 Thermal Cycler, Applied Biosystems, Waltham, MA, USA) and sequencing of the PCR products. The oligonucleotide primers used in this study were forward primer LacbF (50-TGCCTAATACATGCAAGT-30) and reverse primer LacbR (50-CTTGTTACGACTTCACCC-30) [60], obtained from Metabion (Martinsried, Germany).

The PCR analysis was performed in final reaction volumes of 20 µL containing 1 µL of DNA (50 ng), 0.5 µL of each primer and 8 µL of Red-Taq DNA Polymerase Master Mix (Canvax Biotech, S.L., Spain). The parameters of amplification were the following: initial denaturation at 94 °C for 5 min, 35 cycles of 1 min at 94 °C, 45 s at 50 °C and 2 min at 72 °C, and final extension at 72 °C for 5 min. Further, the obtained amplicons were stained with Safe View (NBS Biologicals, Huntingdon, England) and separated on 1% (*w*/*v*) agarose gel carried out in 0.5×Tris-Borate-EDTA (TBE) buffer (45 mmol/L of trisborate and 1 mmol/L of ethylenediaminetetraacetic acid (EDTA)) for 60 min at 100 V, using a VWR Mini Electrophoresis system (VWR, Darmstadt, Germany) and MiniBis Pro (DNR Bio-Imaging Systems, Jerusalem, Israel) for gel visualization. The PCR products (approximately 1450 bp) were cut out from the gel and purified with Clean-Easy™ Agarose Purification Kit (Canvax Biotech, S.L., Spain). Sequencing of the PCR products was performed using MicrosynthSeqlab (Göttingen, Germany).

Sequence similarity search was performed in the GenBank data library using the BLAST algorithm (www.ncbi.nlm.nih.gov/BLAST/ accessed on 28 August 2022). The sequence information was performed using CLUSTAL on MEGA 11 software [61] for assembly and alignment. The 16S rDNA sequences of LAB strains were compared to sequences from type LAB strains held in GenBank (www.ncbi.nlm.nih.gov accessed on 28 August 2022) and recorded with accession number from OP315692–OP315711 from 28 August 2022 (https://www.ncbi.nlm.nih.gov/nuccore/?term=OP315692:OP315711). Nucleotide substitution rates were calculated [62], and phylogenetic tree was constructed through the neighbor-joining method using MEGA 11 software [63].

### 2.5. Screening of Lactic Acid Bacteria for Their Ability to Produce Exopolysaccharides

The strains were initially activated by transferring 1–2 beads from Microbank™ vials into MRS broth and subsequent cultivation at 37 °C for 24 h under anaerobic conditions. The ability of the isolated strains to synthesize exopolysaccharides was tested by using a growth medium for exopolysaccharides synthesis composed of sucrose—20 g/L, K_2_HPO_4_—2 g/L, sodium acetate—5 g/L, triammonium citrate—2 g/L, MgSO_4_—0.2 g/L, MnSO_4_—0.05 g/L, yeast extract—4.0 g/L, Tween 80—1.0 g/L, pH = 5.7 [9]. Sterile sample tubes containing 10 mL of sterile exopolysaccharide-synthesis medium were inoculated with 1 mL of the activated cultures and incubated at 25 °C for 72 h without shaking. After incubation, the biomass was removed from the culture medium through centrifugation at 4428× *g* for 15 min at 15 °C. Two volumes of chilled 96% (*v*/*v*) ethanol were added to the supernatant to precipitate any exopolysaccharides present in the supernatant. All clouded samples were regarded as exopolysaccharide-positive.

### 2.6. Selection of a Carbon Source for Exopolysaccharide Synthesis

#### 2.6.1. Seed Cultures

Seed cultures of six strains (ZS1, ZS3, ZS4, ZS5, ZE2, ZH2) were prepared through two consecutive incubations in MRS broth for 24 h at 37 °C without shaking. The cultures were then used for the experiment with exopolysaccharide production with different carbon sources.

#### 2.6.2. Exopolysaccharide Synthesis

Three variants of the EPS-synthesis culture medium (see Section 2.5) were prepared with different carbon sources, viz., glucose, sucrose and fructose, and each isolate was tested for EPS production in each of the media. These were the main sugars present in the fermented foods used in this study for the isolation of EPS-producing lactic acid bacteria.

Test tubes containing 10 mL of medium were inoculated with 1 mL of the seed culture and incubated without shaking for 72 h at 25 °C. The biomass was then separated through centrifugation at 4428× *g* for 15 min at 15 °C. Trichloroacetic acid (80%, *v*/*v*) was added to the separated supernatant in a volume aiming to achieve a final acid concentration of 4% (*v*/*v*) in the supernatant. The treated supernatant was then left for 24 h under refrigerated conditions (4–8 °C). Afterward, the samples were centrifuged at 3150× *g* for 15 min at 15 °C to separate the precipitated proteins. The supernatant was separated and two volumes of absolute ethanol were added to it. The mixture was maintained again for 24 h under refrigerated conditions (4–8 °C). The samples were then subjected to centrifugation at 3150× *g* for 15 min at 15 °C to separate the precipitated exopolysaccharides. The supernatant was discarded, and 2 mL of water was added to the separated exopolysaccharides and homogenized until it was completely dissolved. The concentration of the exopolysaccharides in the obtained extraction solution was determined spectrophotometrically through the phenol-sulfuric acid method [64].

#### 2.6.3. Determination of Biomass Optical Density

After separation of the biomass from the supernatant, the cells were washed twice with distilled water and the absorbance of the bacterial suspension was measured at a visible wavelength of 660 nm.

### 2.7. Isolation and Purification of Exopolysaccharides

The strain *Lp. plantarum* ZE2 was cultivated in two EPS-synthesis media with glucose and fructose used as carbon sources. Incubation conditions were the same as those in Section 2.5.

#### 2.7.1. Isolation of Exopolysaccharides

The biomass was first separated from the culture medium through centrifugation at 4428× *g* for 15 min at 15 °C. The volume of the obtained supernatant was then reduced fivefold through a vacuum concentration at 50 °C (−0.1 MPa). To precipitate the EPS, 96% (*v*/*v*) cold ethanol was added to the concentrated supernatant (1:3 *v*/*v*) and the mixture was incubated overnight at 4 °C. To recover the precipitate formed during storage, centrifugation at 3150× *g* was performed for 15 min at 15 °C. The precipitate was then dissolved in ultrapure water and subjected to extensive dialysis (MEMBRA-CEL^®^ dialysis tubing SERVA Electrophoresis, Heidelberg, Germany, d = 22 mm, molecular weight cut-off (MWCO) 3500 Da) against distilled water for 72 h at 4 °C, with periodical changing of the water. The dialyzed EPSs were centrifuged at room temperature, filtered through a Büchner funnel and freeze-dried. The first EPS, which was isolated from the culture medium containing glucose as a carbon source, was named EPS-1, while the second one obtained from fructose-containing medium was called EPS-2.

#### 2.7.2. Qualitative Tests for Determination of Monosaccharide Unit in Polysaccharides

A secondary L-cysteine-sulfuric acid color reaction, together with a reaction with resorcinol and hydrochloric acid, was employed as a presumptive qualitative test for the estimation of the types of classes of aldohexoses and/or ketohexoses that comprised the EPS [65].

##### General Analytical Methods

A micro-Kjeldahl method was employed for the estimation of the crude protein content of EPS-1 and EPS-2. The quantification of nitrogen, expressed as the ammonia content of the digested sample, was performed through the acetylacetone–formaldehyde colorimetric method, using ammonium sulfate as a standard [66]. The results were calculated by applying a nitrogen-to-protein conversion factor of 5.7 and a correction for the presence of non-protein nitrogen [67].

The total carbohydrate content of the EPS was measured through the phenol-sulfuric acid reaction using a mixture of glucose and glucosamine (1.5:0.5, *w*/*w*) as a standard [63] Initially, 20 mg of EPS was hydrolyzed with 2 M trifluoroacetic acid (TFA) (10 mL) for 1.5 h at 121 °C to liberate the monosaccharide constituents. Hydrolysates were vacuum-dried at 40 °C and re-dissolved in distilled water. This step was repeated twice to ensure the complete removal of TFA. Finally, the hydrolysates (10 mg/mL) were filtered (0.45 μm-ϕ) and used as samples for analysis. The absorbance was measured at a visible wavelength of 490 nm.

To determine the total uronic acid quantity, an automated [1,1′-biphenyl]-3-ol analysis was conducted using a continuous flow analyzer Skalar San++ system (Skalar Analytical BV, Breda, The Netherlands) according to the instructions of the manufacturer. Absorption was measured at visible wavelength of 530 nm and glucuronic acid (12.5–100.0 μg/mL) was used as a standard.

The qualitative estimation of rare sugars was performed through the periodate-thiobarbituric acid colorimetric method of Karkhanis et al. (1978) [68] as described exhaustively by Ognyanov et al. (2018) [69].

The hexosamine content was analyzed through the 2,4-pentanedione-p-dimethylaminobenzaldehyde method according to the procedure described by Davidson (1966) [70]. Aliquots of TFA-hydrolyzed samples were used for the analysis. In brief, to 1 mL of the hydrolisate, 1 mL of a 2 per cent solution of acetylacetone was added. The tubes were then shaken and left standing for 45 min at 90 °C. After cooling, 2 mL of ethanol and 1 mL of Ehrlich’s reagent were added. After thorough mixing, the color was allowed to develop for 60 min before measuring the absorbances at 540 nm. The calibration curve was prepared by using aliquots of the diluted D-(+)-glucosamine.HCl standard solutions (5 to 25 μg per mL).

The acetyl content of EPS was estimated photometrically through the hydroxamic acid reaction method of McComb and McCready (1957) [71], using β-D-glucose pentaacetate (24–120 µg/mL) as a standard.

##### Monosaccharide Composition Analysis

For the determination of the monosaccharide composition, the EPS was hydrolyzed as described above. Ten microliters of hydrolysate were auto-injected into a Nexera-i LC2040C Plus UHPLC system (Shimadzu Corporation, Kyoto, Japan), coupled with a Zorbax Carbohydrate column (4.6 × 150 mm, 5 μm-ϕ) and Zorbax Reliance Cartridge guard-column operating at 35 °C. The sample was eluted with a mobile phase composed of a mixture of acetonitrile:H2O (80:20, *v*/*v*) at a flow rate of 0.6 mL/min. The eluate was monitored using a refractive index detector (RID)-20A (cell temperature of 35 °C). The concentration of sugars in the sample was deduced using a calibration curve constructed by plotting the peak area (XX-axis) against five different concentrations (YY-axis) for each sugar. The peak corresponding to different sugars in the sample was confirmed through a comparison of the retention time with that of the standards. D(+) Mannose (Man) (99%), L(+) Rhamnose (Rha).H_2_O (99%) and D-Glucuronic acid (GlcA) (≥98+%)—purchased from Alfa Aesar, Thermo Fisher GmbH, Kandel, Germany; and D(+) Xylose (Xyl) (≥99%), D(+) Glucose (Glc) (≥99.5%), D(−) Fructose (Fru) (≥99%) and D(+) Glucosamine hydrochloride (GlcN.HCl)—purchased from Sigma-Aldrich, Merck KGaA, Darmstadt, Germany.

##### Molecular Weight Distribution Analysis

The EPSs (2 mg/mL) were first completely solubilized in distilled water through incubation for 24 h at room temperature. The analysis of EPS was carried out on a Nexera-i LC2040C Plus UHPLC system (Shimadzu Corporation, Kyoto, Japan), coupled with an RID-20A detector, using a Bio SEC-3 column (4.6 × 300 mm, 300 Å, 3 μm-ϕ, Agilent, Santa Clara, CA, United States). Ten microliters of the filtered (0.45 μm-ϕ) solution of the sample were auto-injected and eluted at 30 °C using a mobile phase composed of 150 mM NaH_2_PO_4_ (pH 7.0), employing a flow rate of 0.5 mL/min. Pullulan standards (Shodex standard P-82 kit, Showa Denko, Tokyo, Japan) with molecular weights (MWs) in the range of 0.59 × 104 to 78.8 × 104 g/mol were used for the construction of a logarithm standard curve.

##### Fourier-Transform Infrared (FTIR) Spectroscopy

The FTIR spectra of the EPS samples (2 mg) were recorded in the region of 4000–500 cm^−1^ using the attenuated total reflection technique on Tenzor 27 (Bruker, Rheinstetten, Germany), controlled through OPUS 8.7. software. The two spectra were analyzed in Spectragryph 1.0 software (Dr. Friedrich Menges, file version 1.0.0.0).

### 2.8. Statistics

The HPLC analyses in part “Isolation and purification of exopolysaccharides” were performed in duplicates, whereas the other analyses were run in triplicates. Results are expressed as mean values ± standard deviations (SD).

## 3. Results

### 3.1. Isolation of Lactic Acid Bacteria from Plant-Based Foods

Naturally fermented plant-based foods have been part of the human diet around the world since ancient times and represent a source of many health-beneficial compounds, including the lactic acid bacteria themselves and their metabolites, some of which have a targeted use in the food industry. In the present study, six naturally fermented food products (5 beverages and 1 pickled product) were chosen for the selection of natural lactic acid bacteria—4 samples of Bulgarian Boza, 1 sample of Beninese Degue and 1 sample of sauerkraut prepared according to a traditional Bulgarian method. Preparation technologies of Boza and Degue are described in detail in the literature [72,73]. The choice was based on consideration of the raw materials and the nature of the products to ensure a better chance for isolating EPS-producing lactic acid bacteria.

A total of 43 isolates with typical characteristics of lactic acid bacteria were obtained from six plant-based fermented foods and beverages by plating on MRS agar and M17 agar (Merck KGaA, Darmstadt, Germany).

To prove that the isolates obtained belong to the group of lactic acid bacteria, pure cultures were phenotypically characterized based on colony features, cell morphology, Gram stain and catalase activity. All isolates were found to be Gram-positive and catalase-negative. The results from the characterization of microbial isolates are summarized in Table 1.

The microscopic observation showed that almost half of the isolates had rod-shaped cells (20 isolates) and the remaining 23 isolates were cocci. It is interesting to note that the fermented beverages yielded mostly cocci (18 isolates) and only 5 isolates were rods. In contrast, rod-shaped isolates were predominantly obtained from sauerkraut (15 isolates), and only 5 isolates from this source were cocci.

### 3.2. Molecular Identification of Lactic Acid Bacteria

All obtained isolates were subjected to molecular identification through PCR amplification of the 16S rRNA gene and sequencing of the resulting PCR fragments. The obtained sequences were analyzed through BLAST and identified to the species level. The results are presented in Table 2.

The predominant species found in sauerkraut was *Lactiplantibacillus plantarum* (15 isolates), while 12 isolates from the fermented beverages were identified as *Pediococcus acidilactici*. The other five isolates from sauerkraut were identified as *Pediococcus pentosaceus*. The five fermented beverage products presented a higher variety of LAB: *Limosilactobacillus fermentum* (3 isolates), *Enterococcus faecium* (3 isolates), *Levilactobacillus brevis* (1 isolate), *Lactiplantibacillus plantarum* (1 isolate) and *Pediococcus pentosaceus* (3 isolates).

**Figure 1:**
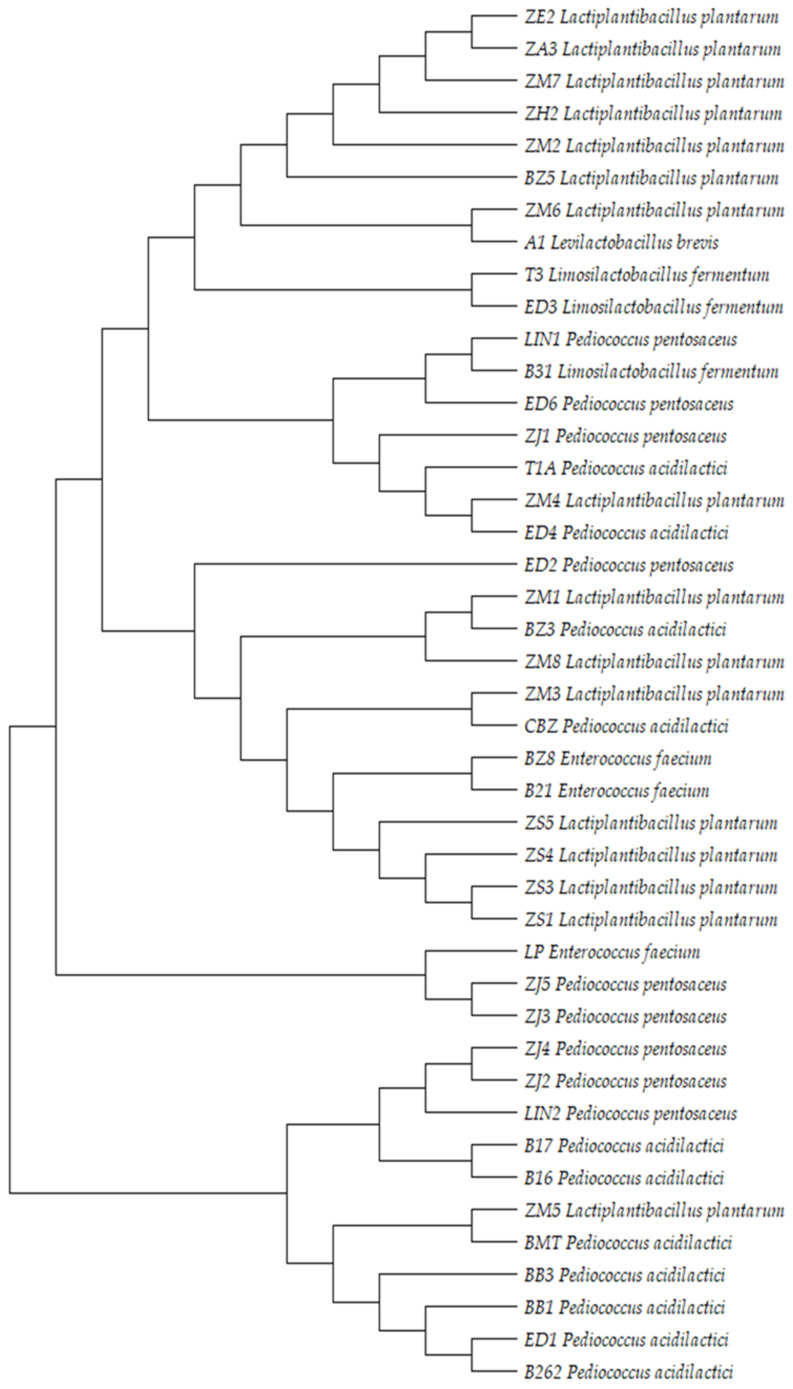
Evolutionary tree constructed through the neighbor-joining method.

The phylogenetic tree presented in Figure 1 shows the relative positions of the 43 lactic acid bacteria strains. As expected, the bacterial strains from the sampled fermented plant-based products include the heterofermentative *Lactiplantibacillus plantarum*, *Pediococcus acidilactici*, *Limosilactobacillus fermentum*, *Enterococcus faecium* and *Pediococcus pentosaceus*.

The Gram-positive rod-shaped *Lactiplantibacillus plantarum* is the most frequently isolated species from sauerkraut [74]. *Lactiplantibacillus plantarum* ZM6 occupies the same column of the phylogenetic tree as *Levilactobacillus brevis* A1. The *Lactiplantibacillus plantarum* strains ZM1, ZM3, ZM5 and ZM4 obtained from sauerkraut occupy the same cluster with the strains of the genus *Pediococcus strains* BMT, CBZ, BZ3 and ED4 obtained from boza. *Enterococcus faecium* strain LP clustered independently of sequence divergence. Surprisingly, in the present study, sauerkraut harbored relatively few isolates of *Levilactobacillus brevis* and only two isolates of *P. pentosaceus*, which are reported by other authors as the major bacterial species involved in sauerkraut fermentation [75].

### 3.3. Screening of Lactic Acid Bacteria for Their Ability to Produce Exopolysaccharides

The 43 identified strains were screened for their ability to produce exopolysaccharides in a liquid exopolysaccharide-synthesis medium.

Temperature is a critical factor in polysaccharide synthesis, with the best results reported at 25 °C, compared to the optimum temperature for growth of lactic acid bacteria [56]. The production of exopolysaccharides by the isolated LAB strains was confirmed by mixing the supernatant of each culture with alcohol. The formation of a precipitate indicated the presence of EPS. As a result of the screening, only six strains (ZS1, ZS3, ZS4, ZS5, ZH2, ZE2) demonstrated the ability to produce exopolysaccharides in varying amounts (Table 3). All of them belonged to the species of *Lactiplantibacillus plantarum* and were isolated from sauerkraut.

### 3.4. Selection of a Carbon Source for Exopolysaccharide Synthesis

As previously mentioned, three different carbon sources (glucose, sucrose and fructose) were tested in order to select the most appropriate for exopolysaccharide synthesis and to explore whether the carbon source affects the composition of the produced EPS. Each carbon source was applied in the same concentration as the optimal glucose level in the standard nutrient medium for cultivation of lactic acid bacteria. Sucrose was clearly the most EPS-productive carbon-source for all of the strains except for ZS1, which showed the best yield from fructose (152.34 mg/L). This effect was most clearly observed for strains ZS3 and ZE2, with ZE2 achieving the highest EPS yield of 211.53 mg/L when sucrose was applied as a carbon source.

With glucose as a carbon source, the highest yield of exopolysaccharide was obtained by the strain ZS3 (178.08 mg/L), while the highest EPS yield from fructose was gained by strain ZS4 (173.24 mg/L). In general, glucose and fructose generated relatively close levels of EPS production, compared to sucrose. It was interesting to find that, compared to glucose, fructose generated higher EPS yields for four of the strains—ZS1, ZS4, ZS5 and ZH2, especially for strain ZS5 where the EPS yield was about 31% higher.

**Figure 2:**
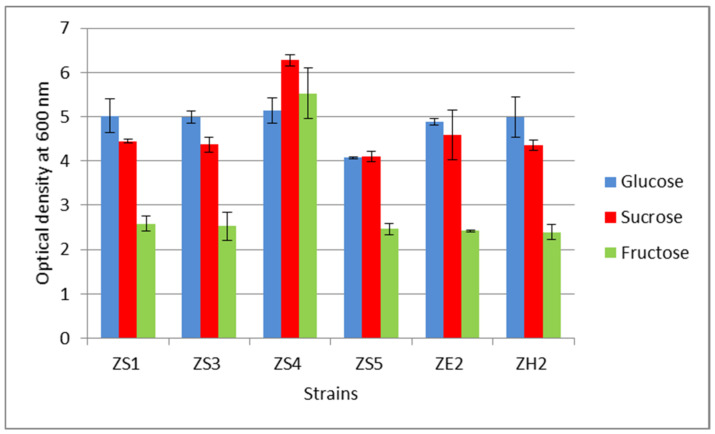
Optical density of biomass obtained in cultivation with different carbon sources, viz., glucose, sucrose and fructose. The error bars represent the standard deviation (*n* = 3).

The effect of the three tested carbon sources on biomass accumulation was also monitored in this study. The amount of biomass obtained was determined by measuring the optical density (OD) at a visible wavelength of 660 nm (Figure 2).

In contrast to EPS production, where sucrose produced the highest yields, in terms of biomass formation, glucose proved to be the most appropriate carbon source. Although *Lp. plantarum* strains generally utilize fructose as a carbon source [76], five of the six tested strains in our study showed the lowest ability for biomass accumulation in the fructose-containing medium, with almost a double difference compared to glucose utilization, except for strain ZS4, which showed good affinity to fructose.

The highest optical density of 6.33 was achieved by strain ZS4 cultivated with sucrose as a carbon source, and the lowest by strain ZH2 with fructose (2.41). Comparison of the results demonstrates that the effect of the carbon source on EPS production is not related to that on biomass accumulation by each strain.

### 3.5. Yield and Chemical Characterization of Exopolysaccharides

Following previous procedures, the next step of this study aimed at the isolation and characterization of the EPS produced by strain *Lactiplantibacillus plantarum* ZE2 in EPS-synthesis culture media with glucose (EPS-1) and fructose (EPS-2) used as a carbon source. Strain *Lactiplantibacillus plantarum* ZE2 was selected for these experiments based on the demonstrated highest EPS production both during the qualitative screening experiment (Table 3) and in the cultivation experiments (Table 4).

Since all six EPS-producing strains were isolated from sauerkraut, and the main sugars in cabbage are glucose and fructose [77], we selected these two monosaccharides to explore in a possibly more distinctive way the effect of the carbon source on the composition of the produced EPS.

After cultivation, the EPS produced in the two culture media were isolated, the yield was determined and the products were subjected to chemical characterization. The results are summarized in Table 5.

Interestingly, the yield of EPS-1 was two times higher than that of EPS-2. The secondary L-cysteine-sulfuric acid color reaction served as a convenient qualitative test for different sugars, giving a green color and suggesting that glucose was the main building block of both EPSs. In addition, based on the fructose-specific test, it became clear that the two EPSs did not contain ketohexoses. Remarkably, both EPSs tested positive for rare sugars (a typical pink color was observed), and a positive test result with 2-thiobarbituric acid reaction suggested the presence of 2-keto-3-deoxy-octonate (KDO), 3-deoxy- and 3,6-dideoxy hexoses. Together with these sugars, it is suggested that sialic acid and N-acetyl-hexosamine were also pink-color-producing constituents after oxidation. It is interesting to note that acetyl groups were found in low amounts, suggesting that some sugar residues were O-acetylated to a different extent. Differences in total carbohydrates were observed as well. EPS-2 was characterized by a higher carbohydrate content of 60% (*w*/*w*). Also interesting is that protein constituted a considerable amount, which was the same in both polymers (41%), indicating that the EPSs were glycoproteins or proteoglycans. The amount of uronic acids was not significant in both EPSs, while neutral sugars represented a large part of the total sugars.

Despite the fact that the EPS-producer ZE2 was cultivated with different carbon sources, the monosaccharide analysis revealed that there were no qualitative differences between the two polymers, and no considerable quantitative differences in the monosaccharide contents. Glucose was the major monosaccharide in both EPSs, amounting to 18.4% and 22.3% (*w*/*w*) in EPS-1 and EPS-2, respectively. They were also composed of lower amounts of mannose, rhamnose and xylose. An interesting finding is that hexosamines were the second most abundant constituent of the two EPSs. Hexosamines in EPS-1 represented an almost two-times-higher content (12.6%) in the polysaccharide composition, compared to EPS-2 (6.7%), since they accounted for nearly 27% of the total sugar content of EPS-1. The presence of acetyl groups in the EPS may be related to the presence of acetylated amino sugars, such as sialic acids and N-acetyl-D-glucosamine. The obtained results show that the two EPS are polymers rich in glucose accompanied by hexosamines and protein. Therefore, the presence of a (hetero)glucan-type PS may be suggested.

#### Molecular Weight Distribution of Exopolysaccharides

**Figure 3:**
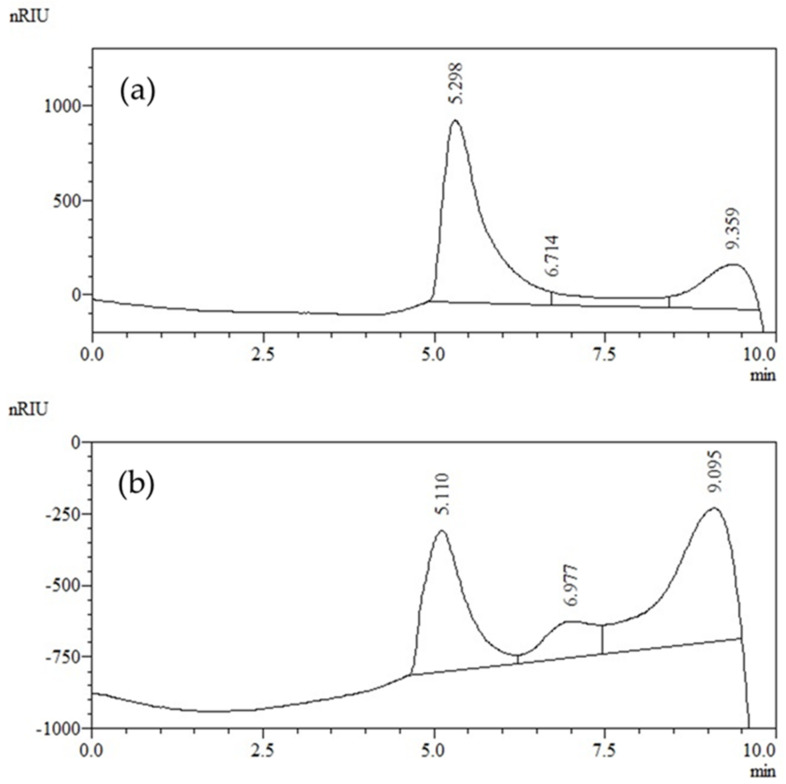
High-pressure size exclusion chromatography (HPSEC) elution pattern of exopolysaccharides (EPS) produced by the strain *Lactiplantibacillus plantarum* ZE2 in growth media containing (**a**) glucose (EPS-1) and (**b**) fructose (EPS-2).

HPSEC was used for the characterization of the two EPS samples (Figure 3). Product EPS-1 was found to elute within one main peak. It covered the mass range between 40 × 104 g/mol and 70 × 104 g/mol. The high-molecular-weight (HMW) fractions (retention time (RT) 5.0–6.0 min) occupied a higher percentage (70%) of the total (100%) peak area, hence the highest percentage of EPS-1. As shown in Figure 3a, sample EPS-1 exhibited a small peak of smaller size, which eluted at an RT of 8.5–9.5 min. This suggested a small amount of EPS-1 fraction (21%) having a mass range between 10 × 104 g/mol and 1 × 104 g/mol.

A significant difference was observed in the elution pattern of EPS-2, depicted in Figure 3b. This sample consisted of three distinct fractions with different distributions of molecular weights and higher heterogeneity. The first main fraction was eluted early, between 4.5 and 6.5 min. It comprised about 36% of the total peak area (100%) and, therefore, represented a smaller percentage of EPS-2. It covered the mass range of 78 × 104 and 20 × 104 g/mol. Next to those fractions, a second peak was eluted (RT 6.5–8.0 min), which represented fractions composed of lower-molecular-weight fragments. Another peak represented a group of lower-molecular-weight fragments (<10 × 104 g/mol), which was eluted between 8.0 and 10 min. The last two peaks occupied 12 and 52% of the total peak area (64%), suggesting that EPS-2 contained a higher proportion of lower-molecular-weight fractions (64%).

**Figure 4:**
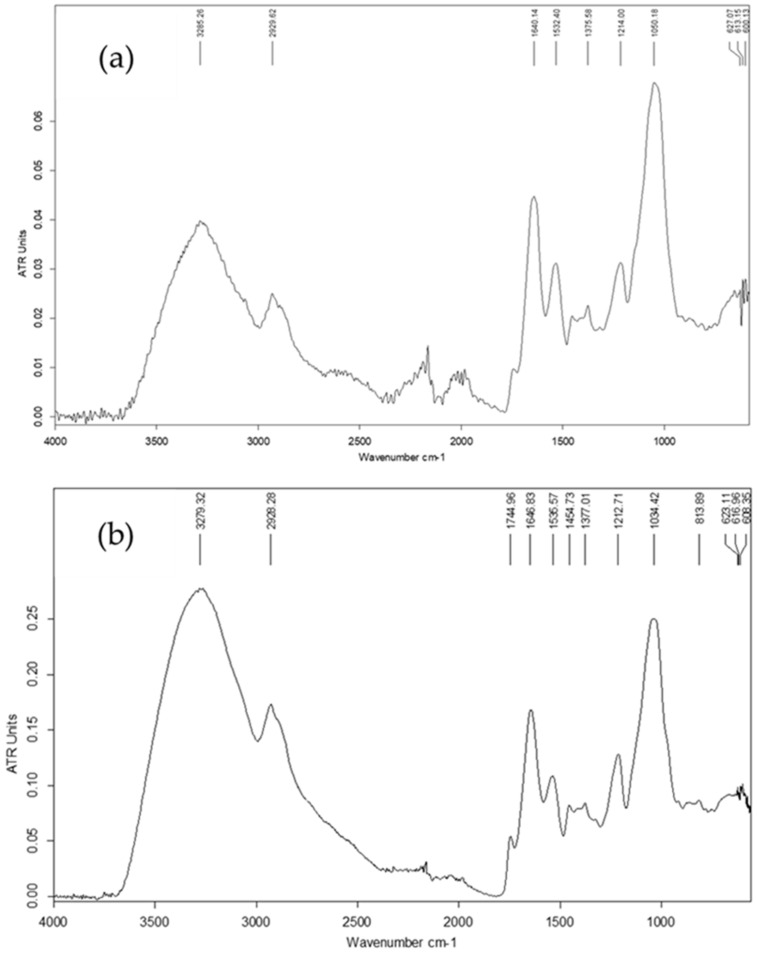
Fourier-transform infrared spectra of exopolysaccharides (EPS) by the strain *Lactiplantibacillus plantarum* ZE2 in growth media containing (**a**) glucose (EPS-1) and (**b**) fructose (EPS-2).

The Fourier-transform infrared spectra of EPS-1 and EPS-2 are shown in Figure 4. There were no significant differences between the spectra of the two polymers, suggesting that there were no differences in the glycosidic bonds or sugar rings of the produced EPSs. The spectra were typical for polysaccharides because a band appeared at about 3280 cm^−1^ and two bands appeared at about 2930 cm^−1^, which were assigned to O–H, C–H and C–H2 stretching, respectively. However, the band at 3340 cm^−1^ due to O–H stretching could not be distinguished from that showing N–H stretching (hydrogen-bonded N–H2 group).

Several bands can be assigned to the amide group vibrations bearing in mind the higher protein content present in the EPS. The bands at 1535 and 1646 cm^−1^ were assigned for δ(N–H) and ν(C–N) of the amide II region and N-linked C=O of amide I structure vibrations [78]. The absorption bands at 1638 and 1411 cm^−1^ could also be attributed to δs(CH2) and δs(CH3) of proteins rather than the ionized carboxyl groups of uronic acids because they were not found in the examined EPS [79]. It was highly unlikely that absorption at 1246 cm^−1^ and a weak band at 1720 cm^−1^ could be attributed to the C=O stretching vibration of the O-acetyl group (ester) and τ(CH2) due to its lower amount (Table 5). However, bands in the region of 1200–1350 cm^−1^ were typical for amide III vibrations (δ(N–H) and ν(C–N)) of protein constituents. The amide III region included N-H in-plane bending coupled with C–N stretching and also indicated C-H and N-H deformation vibrations. The absorption peak at 1535 cm^−1^ corresponded to monosaccharides’ C–OH bending vibration. The bands in the region of 1000–1200 cm^−1^ range indicated that the sugar units comprising the EPS had a pyranose ring. In general, the band at 1375 cm^−1^ assigned to the C–H bending vibration of the CH3 group and ω (CH2) may originate from C–H vibrations in monosaccharide units. The presence of bands in the range of 1200–1000 cm^−1^ indicated the existence of C–O–C and C–O–H bonds, in addition to O–C–O, C–O and C–C stretching of glycosidic bond vibration of glucose-containing polysaccharides. Moreover, the lack of an absorption band at 890 cm^−1^ indicated the absence of a β-glucoside bond in these EPSs.

## 4. Discussion

Spontaneously fermented foods and beverages are a natural source of a wide variety of lactic acid bacteria, producing a number of metabolites with beneficial effects on food quality, as well as human nutrition and health. Exopolysaccharides from lactic acid bacteria are especially valuable due to their texturizing properties in foods and health-promoting potential, and the possibility for in situ production using the fermenting starter cultures [50]. The most frequently isolated EPS-producing lactic acid bacteria are representatives of the genera *Streptococcus, Lactobacillus, Lactococcus, Leuconostoc* and *Pediococcus* [54,56,80]. In the present study, a total of 43 LAB isolates were obtained from plant-based fermented food products—4 traditional Bulgarian fermented products “Boza” (a rye boza, a rye-wheat boza and 2 products of wheat boza), one home-made sauerkraut and a traditional fermented product from Benin—Dèguè made from white sorghum. The isolates were initially subjected to phenotypic characterization. Further, molecular identification of the isolates to the species level was performed through amplification of the 16S rRNA gene and subsequent sequencing of the 16S rDNA. It was found that almost all of the isolated LAB strains were representatives of the genera *Pediococcus* and *Lactobacillus*. From the *Pediococcus* genus, *Pediococcus acidilactici* predominated and this species was found only in the fermented beverage samples. The second found species of this genus, *Pediococcus pentosaceus*, was mainly isolated from sauerkraut (5 out of 8 isolates). Unlike fermented beverages, where cocci-shaped bacteria predominated, the rod-shaped *Lactiplantibacillus plantarum* (formerly *Lactobacillus plantarum*) was the dominant species in the sauerkraut sample. This is one of the most frequently isolated LAB species from sauerkraut and fermented vegetable products in general [80]. Our results confirmed the literature data, as 15 of the 20 sauerkraut isolates belonged to this species.

Screening for exopolysaccharide synthesis was performed with all 43 strains. Although the literature reports on a number of exopolysaccharide-producing strains of the LAB genera found in the present study, the isolates from the *Lactiplantibacillus* genus presented the highest probability of finding EPS producers, since the sauerkraut brine had a viscous consistency suggesting the presence of exopolysaccharides.

Various screening methods for exopolysaccharide producers are available, with the use of both solid and liquid media [81]. Although many authors perform the screening on agar medium, we applied a method using a liquid culture medium described by Oleksy-Sobczak et al. [49], which enables the obtaining of distinctive results. Since the composition of the nutrient medium has a significant influence on EPS synthesis, the advantage of this culture medium is that it is not as rich in nutrients as MRS, which can enhance EPS synthesis even in weaker microbial producers. The combination of the medium composition and cultivation at a lower temperature (25 °C) for a longer period of time (72 h) is proven to contribute to a more intensive EPS synthesis [49]. This can be explained by the fact that exopolysaccharide production is a protective mechanism of bacteria against adverse environmental conditions, such as low or high pH, low or high temperature, and osmotic stress, or against biological factors (e.g., phages) [50]. There are sufficient data on the relationship between microbial stress and the expression of the genes responsible for the synthesis of EPS by lactic acid bacteria [52]. Also, the used culture medium is colorless, which allows for easier detection of EPS production. Also, the use of this nutrient medium is more appropriate if the weight method is further applied for quantification of the produced EPS, since when working with the MRS medium, some of its compounds will also be deposited on the filter and will compromise the final result. Moreover, the EPS-synthesis culture medium is significantly cheaper than MRS [49].

The choice of cultivation temperature and pH was also based on the literature data. Many authors define temperature as a critical factor for EPS production and, in most cases, the range of 20–25 °C was found the most suitable for cultivating exopolysaccharide producers [53,81,82]. Temperatures below 20 °C are not economically feasible, since the costs of cooling during the production process are higher compared to a process where heating is necessary.

The choice of pH 5.7 for the culture medium was based on the commonly used MRS broth, which has the same pH value. There are also data that the most suitable pH for exopolysaccharide synthesis is around 6.0, although species- and strain-variability has been observed for the optimal levels of both temperature and pH [48,83,84].

Our analysis showed that only 6 of all 43 strains were able to produce exopolysaccharides. This is not unusual, given that other authors also report a small percentage of EPS producers in screening studies [85,86]. As expected, given the viscous consistency of the sauerkraut brine, all six EPS-producing strains were isolated from sauerkraut and belonged to *Lactiplantibacillus plantarum*. This was not surprising, since, in general, the genus *Lactiplantibacillus* is reported to produce the highest EPS yields [87,88]. Also, exopolysaccharides obtained from this LAB species attract high interest among researchers and industry due to their technological properties in foods, biological activity and health benefits [86].

Along with temperature, the carbon source in the growth medium is a key factor for EPS synthesis, affecting not only the yield, but also the chemical composition of the synthesized exopolysaccharides [89]. Therefore, the next step of this study was to explore the effect of different carbon sources on EPS production by the selected LAB strains. Glucose and sucrose are the most commonly used carbon sources to obtain significant EPS yields [81]. However, the main sugars in cabbage, which was the source of the six EPS-producing strains, are known to be glucose and fructose [77]. Therefore, the experiment on EPS-synthesis was performed with sucrose, glucose and fructose.

The obtained interesting results showed that glucose and fructose generally yielded similar EPS levels with five of the tested LAB strains. Strain ZS5 represented an exception, generating an EPS yield about 31% higher on fructose compared to glucose as a carbon source.

The highest EPS concentration of 211.53 mg/L was achieved with strain ZE2 when cultivated in the sucrose-containing medium. The levels of exopolysaccharides produced from sucrose varied from 128.91 to 211.53 mg/L; for glucose, the EPS levels achieved were from 107.90 to 168.35 mg/L; and fructose yielded 141.78 to 173.24 mg/L. Strain-based differences were also observed. Strain ZS5 yielded almost 69% higher EPS levels from sucrose compared to glucose, and for strain ZE2, EPS production from sucrose was 33% higher compared to glucose. In general, sucrose was assimilated most efficiently by most of the tested strains, followed by glucose. The difference in carbon source effect on EPS production by the remaining strains was within 10%. Interestingly, one strain—ZS1— showed the highest EPS production from fructose, compared to the other two sugars, yielding 152.34 mg/L of EPS.

Our results are in agreement with those reported by other authors who obtained 10 to 400 mg/L of EPS under non-optimized conditions. However, it is important to note that EPS yield depends not only on strain specificity and the carbon source used, but also on the medium composition and cultivation conditions. Bomfim et al. (2020) [11] found that an *Lp. plantarum* strain was the best EPS producer compared to other LAB strains grown on MRS control medium (glucose) and in MRS supplemented with fructooligosaccharides (FOS). In the glucose-containing MRS medium, the total EPS yield was 378 mg/L for *Lp. plantarum* CNPC003, while in the FOS-containing MRS medium, the yield reached 568.4 mg/L. Li et al. (2014) [90] also cultivated *Lactobacillus* strains on MRS medium, achieving EPS levels of 290.17 mg/L from strain *Lp. plantarum* R315, and Midik et al. (2020) [57] reported an EPS production of up to 550 mg/L from *Lactiplantibacillus plantarum*. The highest EPS values found in the literature were 2.8 g/L reported for *Lacticaseibacillus rhamnosus* RW9595M [58].

In addition to exopolysaccharide production, the influence of the carbon source on biomass accumulation was also investigated. Unlike EPS production, where sucrose was found most suitable for high yields, glucose proved to be the most appropriate carbon source for biomass accumulation for all strains except for ZS4, which gave the highest optical density values when assimilating sucrose.

*Lp. plantarum* species is generally known to be able to utilize fructose as a carbon source, but glucose is much easier metabolized. This was also demonstrated in our study. Five of the six tested strains showed the highest biomass accumulation in the glucose-containing medium, with almost a double difference compared to fructose utilization, except for strain ZS4, which showed a good affinity to fructose and the highest optical density values when assimilating sucrose. However, this strain cannot be attributed to the group of fructophilic lactic acid bacteria, since they are described by an optimum growth on fructose as a carbon source and very poor growth on glucose [91], which is not the case.

Our results from the two experiments show that the effect of the carbon source on EPS production is not related to that on biomass accumulation by each strain. Therefore, strain specificity and the aim of cultivation (biomass or EPS production) must be taken into account when choosing the carbon source.

The chemical composition and molecular weight distribution of LAB-produced EPS are of key importance for their biological activity and functional properties. EPSs are characterized by complex composition, with carbohydrates and proteins usually being the major components of EPS. This was confirmed in our study, as we found that glucose (80–83 mol%) and protein (41% *w*/*w*) predominated in both analyzed EPSs. However, it should be noted that the composition of EPS largely depends on the LAB strain and the culture medium. Kowsalya et al. (2023) [92] reported that the EPS produced by two *Lp. plantarum* strains comprised mainly glucose, galactose and mannose. A study of Saif and Sakr (2020) [93] also showed that glucose was the major sugar constituent of *Lp. plantarum* 47FE-excreted EPS. In contrast to our study, Wang et al. (2017) [94] reported that *Lp. plantarum*-produced EPS was a hetero-polysaccharide mainly composed of mannose, while glucose, galactose and arabinose were minor constituents. Similar to this research team, we did not find significant quantities of uronic acids. Based on these data, it is clear that even within the same LAB species, EPS composition varies significantly due to medium composition (carbon and nitrogen source) and culture conditions, as well as the applied EPS extraction methods.

## 5. Conclusions

The present study was related to the high research interest on the selection of EPS-producing lactic acid bacteria in view of their potential applications in the food and pharmaceutical industry. Forty-three strains were isolated from different plant-based fermented foods (5 cereal-based beverages and 1 sauerkraut product) and identified through molecular methods. The species found were *Pediococcus acidilactici*, *Limosilactobacillus fermentum*, *Pediococcus pentosaceus*, *Enterococcus faecium*, *Lactiplantibacillus plantarum* and *Levilactobacillus brevis*, with a distinctive specificity of distribution according to the food source. Six *Lactiplantibacillus plantarum* strains isolated from sauerkraut showed the ability to produce exopolysaccharides. The effect of the carbon source on EPS and biomass accumulation by the tested strains was further explored by using glucose, fructose and sucrose. In general, sucrose was the best carbon source for EPS production by most of the strains, yielding up to 211.53 mg/L by strain ZE2, while biomass accumulation reached the highest levels in the glucose-based culture medium. Strain ZS4 was an exception, yielding the highest optical density of 6.27 with sucrose. Most strains produced similar levels of EPS with glucose and fructose, with differences of about 10%. In contrast, fructose was utilized more poorly for biomass production, yielding almost 50% of biomass compared to glucose for most strains. The results from these experiments demonstrate that the effect of the carbon source on EPS production is different from that on biomass accumulation by each strain. Therefore, strain specificity and the aim of cultivation (biomass or EPS production) must be taken into account when choosing the carbon source.

The effect of glucose and fructose on the EPS composition was also tested by cultivating strain *Lactiplantibacillus plantarum* ZE2. The yield of EPS-1 (glucose medium) was twice higher than that of EPS-2 (fructose medium), and differences in the levels of all detected sugars were found, which demonstrates that even for the same strain, EPS yield and composition vary depending on the carbon source. Glucose (80–83 mol%) and protein (41% *w*/*w*) were still predominant in both produced EPSs.

The use of LAB cultures for in situ production of EPS may be employed in the food industry to improve product quality and to meet consumer demand for health-promoting functional foods. However, the low yield and the variation in the production capacity of EPS-producing strains still present a limitation for industrial applications. Therefore, careful starter culture selection based on the connection between the carbon source, cultivation parameters and the characteristics of the obtained EPS, which affect their technological effects and biological activity, is key for successful industrial development. On the other hand, EPS production may not be a desirable feature of lactic acid bacteria applied in some food fermentations, since their accumulation in the product deteriorates the quality and is perceived as food spoilage—sauerkraut is a typical example. These implications stress the importance of also exploring the mechanisms leading to EPS production by lactic acid bacteria in different food matrices.

## Figures and Tables

**Table 1:** Lactic acid bacteria isolates obtained from plant-based fermented foods.

No.	Isolate Index	Cell Shape	Gram	Catalase	Food Source
1	BB1	Cocci	Gram (+)	Catalase (−)	Rye Boza
2	BB3	Cocci	Gram (+)	Catalase (−)	
3	T1A	Cocci	Gram (+)	Catalase (−)	
4	T3	Rods	Gram (+)	Catalase (−)	
5	ED1	Cocci	Gram (+)	Catalase (−)	Wheat Boza
6	ED2	Cocci	Gram (+)	Catalase (−)	
7	ED3	Rods	Gram (+)	Catalase (−)	
8	ED4	Cocci	Gram (+)	Catalase (−)	
9	ED6	Cocci	Gram (+)	Catalase (−)	
10	LP	Cocci	Gram (+)	Catalase (−)	Wheat Boza Bena
11	LIN1	Cocci	Gram (+)	Catalase (−)	
12	LIN2	Cocci	Gram (+)	Catalase (−)	
13	BZ3	Cocci	Gram (+)	Catalase (−)	Rye-Wheat Boza
14	BZ8	Cocci	Gram (+)	Catalase (−)	
15	BZ5	Rods	Gram (+)	Catalase (−)	
16	A1	Rods	Gram (+)	Catalase (−)	
17	BMT	Cocci	Gram (+)	Catalase (−)	
18	CBZ	Cocci	Gram (+)	Catalase (−)	
19	B17	Cocci	Gram (+)	Catalase (−)	Beninese White Sorghum Degue
20	B31	Rods	Gram (+)	Catalase (−)	
21	B16	Cocci	Gram (+)	Catalase (−)	
22	B21	Cocci	Gram (+)	Catalase (−)	
23	B262	Cocci	Gram (+)	Catalase (−)	
24	ZM1	Rods	Gram (+)	Catalase (−)	Sauerkraut
25	ZM2	Rods	Gram (+)	Catalase (−)	
26	ZM3	Rods	Gram (+)	Catalase (−)	
27	ZM4	Rods	Gram (+)	Catalase (−)	
28	ZM5	Rods	Gram (+)	Catalase (−)	
29	ZM6	Rods	Gram (+)	Catalase (−)	
30	ZM7	Rods	Gram (+)	Catalase (−)	
31	ZM8	Rods	Gram (+)	Catalase (−)	
32	ZJ1	Cocci	Gram (+)	Catalase (−)	
33	ZJ2	Cocci	Gram (+)	Catalase (−)	
34	ZJ3	Cocci	Gram (+)	Catalase (−)	
35	ZJ4	Cocci	Gram (+)	Catalase (−)	
36	ZJ5	Cocci	Gram (+)	Catalase (−)	
37	ZS1	Rods	Gram (+)	Catalase (−)	
38	ZS3	Rods	Gram (+)	Catalase (−)	
39	ZS4	Rods	Gram (+)	Catalase (−)	
40	ZS5	Rods	Gram (+)	Catalase (−)	
41	ZA3	Rods	Gram (+)	Catalase (−)	
42	ZH2	Rods	Gram (+)	Catalase (−)	
43	ZE2	Rods	Gram (+)	Catalase (−)	

**Table 2:** Molecular identification of lactic acid bacteria from fermented plant-based foods.

No.	Strain Index	Species	Similarity, %	Food Source
1	BB1	*Pediococcus acidilactici*	99.35	Rye boza
2	BB3	*Pediococcus acidilactici*	99.47	Rye boza
3	T1A	*Pediococcus acidilactici*	98.48	Rye boza
4	T3	*Limosilactobacillus fermentum*	99.49	Rye boza
5	ED1	*Pediococcus acidilactici*	99.32	Wheat Boza
6	ED2	*Pediococcus pentosaceus*	99.61	Wheat Boza
7	ED3	*Limosilactobacillus fermentum*	99.83	Wheat Boza
8	ED4	*Pediococcus acidilactici*	99.66	Wheat Boza
9	ED6	*Pediococcus pentosaceus*	100.00	Wheat Boza
10	LP	*Enterococcus faecium*	100.00	Wheat Boza Bena
11	LIN1	*Pediococcus pentosaceus*	99.22	Wheat Boza Bena
12	LIN2	*Pediococcus acidilactici*	99.48	Wheat Boza Bena
13	BZ3	*Pediococcus acidilactici*	99.40	Rye-Wheat Boza
14	BZ8	*Enterococcus faecium*	99.49	Rye-Wheat Boza
15	BZ5	*Lactiplantibacillus plantarum*	99.40	Rye-Wheat Boza
16	A1	*Levilactobacillus brevis*	100.00	Rye-Wheat Boza
17	BMT	*Pediococcus acidilactici*	99.50	Rye-Wheat Boza
18	CBZ	*Pediococcus acidilactici*	98.83	Rye-Wheat Boza
19	B17	*Pediococcus acidilactici*	97.9	Beninese White Sorghum Degue
20	B31	*Limosilactobacillus fermentum*	99.78	Beninese White Sorghum Degue
21	B16	*Pediococcus acidilactici*	96.85	Beninese White Sorghum Degue
22	B21	*Enterococcus faecium*	99.56	Beninese White Sorghum Degue
23	B262	*Pediococcus acidilactici*	97.79	Beninese White Sorghum Degue
24	ZM1	*Lactiplantibacillus plantarum*	99.91	Sauerkraut
25	ZM2	*Lactiplantibacillus plantarum*	99.57	Sauerkraut
26	ZM3	*Lactiplantibacillus plantarum*	99.83	Sauerkraut
27	ZM4	*Lactiplantibacillus plantarum*	99.56	Sauerkraut
28	ZM5	*Lactiplantibacillus plantarum*	100.00	Sauerkraut
29	ZM6	*Lactiplantibacillus plantarum*	99.83	Sauerkraut
30	ZM7	*Lactiplantibacillus plantarum*	99.40	Sauerkraut
31	ZM8	*Lactiplantibacillus plantarum*	99.4	Sauerkraut
32	ZJ1	*Pediococcus pentosaceus*	99.66	Sauerkraut
33	ZJ2	*Pediococcus pentosaceus*	99.58	Sauerkraut
34	ZJ3	*Pediococcus pentosaceus*	99.66	Sauerkraut
35	ZJ4	*Pediococcus pentosaceus*	99.28	Sauerkraut
36	ZJ5	*Pediococcus pentosaceus*	99.74	Sauerkraut
37	ZS1	*Lactiplantibacillus plantarum*	98.40	Sauerkraut
38	ZS3	*Lactiplantibacillus plantarum*	99.66	Sauerkraut
39	ZS4	*Lactiplantibacillus plantarum*	99.08	Sauerkraut
40	ZS5	*Lactiplantibacillus plantarum*	99.66	Sauerkraut
41	ZA3	*Lactiplantibacillus plantarum*	100.00	Sauerkraut
42	ZH2	*Lactiplantibacillus plantarum*	99.24	Sauerkraut
43	ZE2	*Lactiplantibacillus plantarum*	99.77	Sauerkraut

**Table 3:** Screening of exopolysaccharide-producing lactic acid bacteria from plant-based fermented foods and beverages.

№	Strain	Species	EPS Production	No.	Strain	Species	EPS Production
1	BB1	*Pediococcus acidilactici*	ND	23	B262	*Pediococcus acidilactici*	ND
2	BB3	*Pediococcus acidilactici*	ND	24	ZM1	*Lactiplantibacillus plantarum*	ND
3	T1A	*Pediococcus acidilactici*	ND	25	ZM2	*Lactiplantibacillus plantarum*	ND
4	T3	*Limosilactobacillus fermentum*	ND	26	ZM3	*Lactiplantibacillus plantarum*	ND
5	ED1	*Pediococcus acidilactici*	ND	27	ZM4	*Lactiplantibacillus plantarum*	ND
6	ED2	*Pediococcus pentosaceus*	ND	28	ZM5	*Lactiplantibacillus plantarum*	ND
7	ED3	*Limosilactobacillus fermentum*	ND	29	ZM6	*Lactiplantibacillus plantarum*	ND
8	ED4	*Pediococcus acidilactici*	ND	30	ZM7	*Lactiplantibacillus plantarum*	ND
9	ED6	*Pediococcus pentosaceus*	ND	31	ZM8	*Lactiplantibacillus plantarum*	ND
10	LP	*Enterococcus faecium*	ND	32	ZJ1	*Pediococcus pentosaceus*	ND
11	LIN1	*Pediococcus pentosaceus*	ND	33	ZJ2	*Pediococcus pentosaceus*	ND
12	LIN2	*Pediococcus acidilactici*	ND	34	ZJ3	*Pediococcus pentosaceus*	ND
13	BZ3	*Pediococcus acidilactici*	ND	35	ZJ4	*Pediococcus pentosaceus*	ND
14	BZ8	*Enterococcus faecium*	ND	36	ZJ5	*Pediococcus pentosaceus*	ND
15	BZ5	*Lactiplantibacillus plantarum*	*ND*	37	ZS1	*Lactiplantibacillus plantarum*	++
16	A1	*Levilactobacillus brevis*	*ND*	38	ZS3	*Lactiplantibacillus plantarum*	+
17	BMT	*Pediococcus acidilactici*	*ND*	39	ZS4	*Lactiplantibacillus plantarum*	+++
18	CBZ	*Pediococcus acidilactici*	*ND*	40	ZS5	*Lactiplantibacillus plantarum*	++
19	B17	*Pediococcus acidilactici*	*ND*	41	ZA3	*Lactiplantibacillus plantarum*	ND
20	B31	*Limosilactobacillus fermentum*	*ND*	42	ZH2	*Lactiplantibacillus plantarum*	++
21	B16	*Pediococcus acidilactici*	*ND*	43	ZE2	*Lactiplantibacillus plantarum*	+++
22	B21	*Enterococcus faecium*	*ND*				

Note: +—minimal amount; ++—average amount; +++—high amount; ND—EPS not detected.

**Table 4:** Exopolysaccharide concentration (mean values ± standard deviations) obtained through cultivation in culture media with different carbon sources.

Strain	EPS Concentration, mg/L		
	Glucose	Sucrose	Fructose
ZS1	145.81 ± 16.92	128.91 ± 55.85	152.34 ± 15.63
ZS3	178.08 ± 2.86	193.06 ± 19.30	168.81 ± 32.23
ZS4	168.35 ± 18.74	182.74 ± 2.60	173.24 ± 41.69
ZS5	107.9 ± 41.74	181.93± 14.13	141.78 ± 49.07
ZE2	159.23 ± 6.33	211.53 ± 16.50	147.58 ± 25.57
ZH2	154.6 ± 17.89	171.95 ± 13.51	161.18 ± 6.14

**Table 5:** Characterization of the composition of exopolysaccharides produced by the strain *Lactiplantibacillus plantarum* ZE2 from different carbon sources (glucose (EPS-1) and fructose (EPS-2)).

	ЕPS-1	EPS-2
Yield (g EPS/L) ^1^	0.73	0.36
Total carbohydrates ^2^, *w*/*w*%	46 ± 0.6	60 ± 1.0
Rhamnose	1.8 ± 0.3 (8.8) ^3^	2.1 ± 0.1 (8.4)
Mannose	1.1 ± 0.1 (5.6)	1.0 ± 0.05 (4.6)
Glucose	18.4 ± 0.1 (80)	22.3 ± 0.4 (83)
Xylose	1.2 ± 0.2 (5.6)	1.0 ± 0.1 (4.2)
Uronic acids	<0.5 (0)	<0.5 (0)
Hexosamines	12.6 ± 0.1	6.7 ± 0.2
Rare sugars test	+	+
Acetyl content, % (*w*/*w*)	0.7 ± 0.05	0.8 ± 0.0
Protein, % (*w*/*w*) (N × 5.7)	41 ± 0.5	41 ± 0.7

Note: ^1^ A liter of cultural liquid; ^2^ Glc:GlcN equivalent; ^3^ Values in brackets represent the monosaccharide composition in molar percentage. Values for yields are the average of two replicates ± standard deviation (SD), while the other values are the average of 3 replicates ± SD value.

## Data Availability

Data is contained within the article.

## Abbreviations

Carbon dioxide (CO_2_), Colony-forming unit (CFU), de Man, Rogosa and Sharpe (MRS), EPS isolated from the culture medium containing fructose as a carbon source (EPS-2), EPS isolated from the culture medium containing glucose as a carbon source (EPS-1), Ethylenediaminetetraacetic acid (EDTA), European Food Safety Authority (EFSA), European Union (EU), Exopolysaccharide (EPS), Food and Drug Administration (FDA), Fourier-transform infrared (FTIR), Fructooligosaccharide (FOS), Generally regarded as safe (GRAS), Glucose (Glu), Glucuronic acid (GlcA), High molecular weight (HMW), High-pressure size exclusion chromatography (HPSEC), 2-Keto-3-deoxy-octonate (KDO), Lactic acid bacteria (LAB), Mannose (Man), Molecular weight (MW), Molecular weight cut-off (MWCO), Optical density (OD), Polymerase chain reaction (PCR), Qualified Presumption of Safety (QPS), Refractive index detector (RID), Retention time (RT), Rhamnose (Rha), Standard deviation (SD), Trifluoroacetic acid (TFA), Tris-borate-EDTA (TBE), Xylose (Xyl).
